# Orchestrating glucose metabolism: PFKFB2 as a signal-integrating conductor in homeostasis and disease

**DOI:** 10.1016/j.jbc.2026.113294

**Published:** 2026-06-25

**Authors:** Kylene M. Harold, Wan Hee Yoon, Kenneth M. Humphries

**Affiliations:** 1 Department of Biochemistry and Physiology, University of Oklahoma Health Sciences Center, Oklahoma, USA; 2 Aging and Metabolism Research Program, Oklahoma Medical Research Foundation, Oklahoma, USA

**Keywords:** ancillary pathways, fructose 2,6-bisphosphate, glucose metabolism, glycolysis, glycolytic regulation, metabolic regulation, phosphofructokinase

## Abstract

As a bifunctional enzyme, phosphofructokinase-2/fructose 2,6-bisphosphatase (PFKFB or PFK-2) produces and degrades fructose-2,6-bisphosphate (Fru-2,6-P_2_). Because Fru-2,6-P_2_ is a strong allosteric activator of glycolysis, PFKFB is critical to glycolytic regulation. Four isoenzymes of PFKFB have been identified (PFKFB1-4). PFKFB2 is considered the cardiac isoenzyme and is distinct among the isoforms because of its complex regulation *via* multi-site phosphorylation. It plays critical roles in cardiac physiological responses to stress, with its loss a key driver of pathophysiology in metabolic cardiac diseases. However, PFKFB2 is also expressed in multiple additional tissues and is involved in noncardiac pathologies including cancer. Therefore, an ongoing area of research is the regulation of PFKFB2 activity and abundance. Here, we review the history and present knowledge of the structure, function, tissue distribution, and roles of PFKFB2 in physiology, stress response, and pathophysiology, both in the heart and other tissues systemically.

Phosphofructokinase-2/fructose 2,6-bisphosphatases (PFKFBs) are capable of both phosphorylating fructose 6-phosphate (Fru-6-P) to produce fructose 2,6-bisphosphate (Fru-2,6-P_2_) and dephosphorylating Fru-2,6-P_2_ to produce Fru-6-P (1, 2). Fru-2,6-P_2_ is the most potent allosteric activator of phosphofructokinase 1 (PFK-1), which regulates the rate-limiting step of glycolysis (3). PFKFB2 has been deemed the cardiac isoenzyme of the PFKFB family of proteins. Among PFKFB isoenzymes, PFKFB2 is the only one that is known to be regulated at multiple different phosphorylation sites in mammals (2). When dephosphorylated, PFKFB2 acts as a phosphatase, dephosphorylating Fru-2,6-P_2_ and decreasing allosteric activation of PFK-1 (Fig. 1). Conversely, when phosphorylated at its C-terminus in response to local or systemic factors such as adrenergic stimulation, hypoxia, or insulin signaling, PFKFB2 takes a kinase form, producing Fru-2,6-P_2_ and robustly stimulating glycolysis (Fig. 1). By this mechanism, PFKFB2 serves a critical role in the dynamic coupling of cellular stress, insulinemic, or energetic statuses to compensatory glycolytic upregulation. Simultaneously, the positioning of PFKFB2 downstream of major branch points for ancillary glycolytic pathways supports a broader role in glucose metabolism beyond glycolysis alone (Fig. 1).

**Figure 1:**
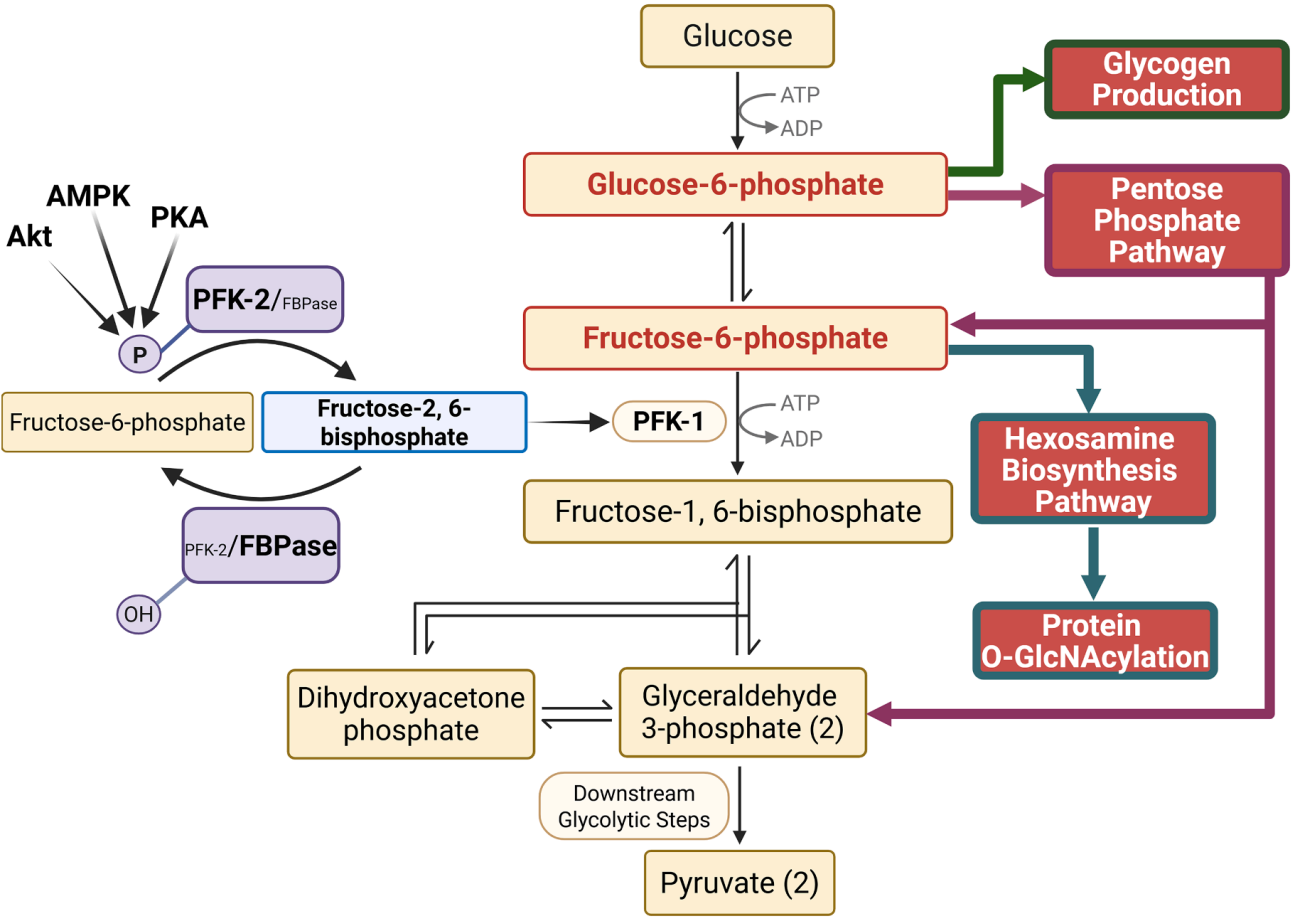
**Role and regulation of PFKFB2 in glycolysis and ancillary pathways.** Regulation of PFKFB2 and its product fructose 2,6 bisphosphate, as well as their roles in regulating metabolite flow into glycolysis, glycogen production, the pentose phosphate pathway, and the hexosamine biosynthesis pathway.

Although initially characterized in the heart, PFKFB2 is now recognized to function in a broader range of tissues and physiological contexts, including metabolic stress, hypoxia, inflammation, and disease states such as diabetes and cancer. However, its precise regulation, tissue-specific functions, and distinction from other PFKFB isoenzymes are incompletely resolved. This is particularly true of its position within various signaling networks and metabolic settings. These uncertainties motivate a comprehensive synthesis of current knowledge regarding PFKFB2 structure, regulation, and function, as well as spaces necessitating further investigation.

In this review, we integrate current knowledge of PFKFB2 with an emphasis on its structural features, regulatory mechanisms, and context-dependent roles in both normal physiology and pathology. We highlight emerging themes across tissues and disease states. Central goals of this review are to clarify how PFKFB2 contributes to metabolic flexibility and stress adaptation, and to identify key gaps that will guide future investigation.

## History and background

The discovery of Fru-2,6-P_2_ in 1980 spurred deeper investigation into the source and regulation of this highly potent stimulator of hepatic glycolysis and regulator of gluconeogenesis (4, 5). From its initial identification and purification in liver, the abundance of this low-molecular-weight PFK-1 regulatory molecule seemed to be sensitive to glucose and glucagon levels (5, 6). However, while determined to be regulated by phosphorylation and dephosphorylation, the exact identity and regulation of the corresponding kinase and phosphatase remained to be elucidated (7). The following year, in 1981, the kinase which produced Fru-2,6-P_2_ from Fru-6-P (phosphofructokinase-2) was identified (8, 9). Upon isolation of the phosphatase which degraded Fru-2,6-P_2_ (fructose 2,6 bisphosphatase) in 1982, it was also noted that the phosphatase co-purified with phosphofructokinase-2 (10). Therefore, it followed naturally that the following year, the catalytic activities responsible for both the production and degradation of Fru-2,6-P_2_ were found to occur on the same polypeptide, as regulated by phosphorylation (11, 12, 13, 14, 15). This marked the discovery of liver phosphofructokinase-2/fructose 2,6-bisphosphatase, which would later be known as the liver splice variant of PFKFB1.

**Figure 2:**
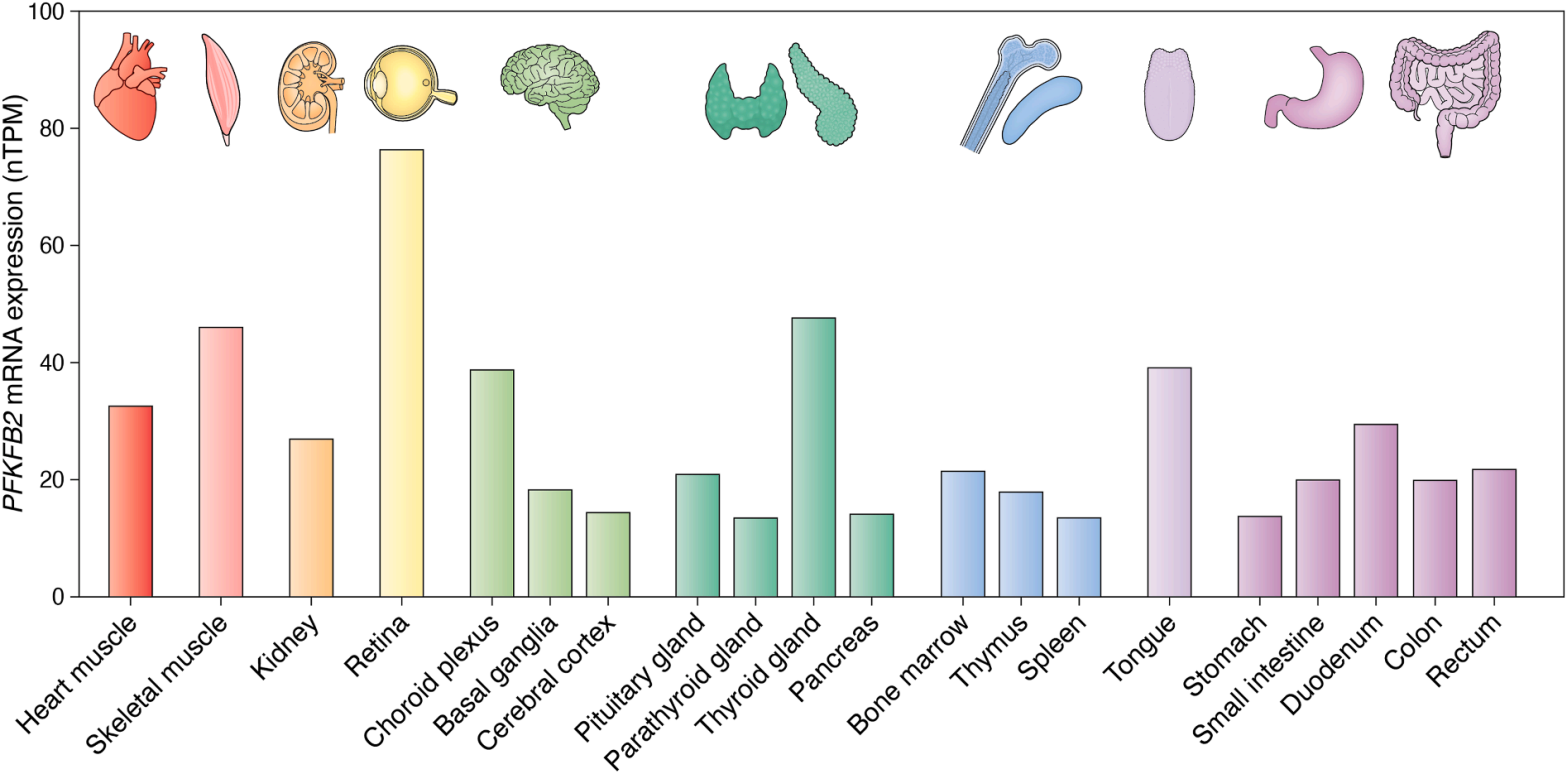
**Top 20 tissues with highest RNA expression from the human protein atlas.** mRNA expression in tissues with highest RNA expression as depicted in the Human Protein Atlas, shown in normalized transcripts per million (nTPM).

Accordingly, studies leading up to this point predominantly focused on the liver, though it was understood that the role of Fru-2,6-P_2_ may extend to other tissues, and/or be governed by more than one PFKFB isoform. Indeed, it was discovered soon after that multiple genes, each located on different chromosomes, encode related yet distinct PFKFB isoenzymes. Initially denoted A-D, the nomenclature PFKFB1-4 was eventually adopted. Through a series of genomic, cloning, and characterization experiments, PFKFB isoenzymes were identified in liver and skeletal muscle (PFKFB1) (12, 13, 16, 17, 18, 19, 20), heart (PFKFB2) (21, 22, 23, 24, 25), testis (PFKFB4) (26, 27), and a ubiquitous inducible isoform identified in the brain (PFKFB3) (28). While these isoenzymes tend to be enriched in these tissues from which they were initially purified, they are not exclusively localized to these tissues (29). For instance, PFKFB2, while initially purified from the heart, exhibits transcript expression in a diverse array of tissues (Fig. 2). Though, it is important to note that post-transcriptional and post-translational regulation play key roles in PFKFB expression, as will be discussed herein. For example, research from our lab has shown PFKFB2 protein levels are significantly decreased in the diabetic heart without corresponding changes in transcript levels (30). Also in the instance of PFKFB2, the kidney exhibits stronger expression of a variant which arises from alternative splicing and is distinct from the heart isoforms (24, 31). Ultimately, these isoenzymes, and the various isoforms thereof, differ not only in tissue expression patterns, but also in kinetic and regulatory properties.

PFKFB2 was first identified and purified from bovine heart (25, 32). Characterization in rat heart followed soon after (21). Subsequent studies revealed homologous activity in mouse kidney, which was identified as a splice variant of PFKFB2 distinct from the cardiac isoform, as previously mentioned (31). The human PFKFB2 gene was later cloned and sequenced, demonstrating strong sequence homology with other mammalian heart PFKFB2 orthologs (22). These early studies laid the foundation for deeper investigations into function, variants, and regulatory mechanisms.

## Structure of PFKFB2

### Protein

In 1990, a foundational study cloned the complementary DNA (cDNA) encoding bovine cardiac PFKFB2 and deduced the amino acid sequence (25). The sequence comprised 530 amino acids, giving a calculated molecular weight of 60.7 kDa for each of the two polypeptide subunits (25). Through this analysis, the study additionally identified key phosphorylation sites Ser 466 and Thr 475 on the C-terminal regulatory portion of the heart enzyme (25). At the time, this marked a unique distinction from PFKFB1 which exhibits phosphorylation at the N-terminus (16). Additional phosphorylation sites have since been discovered, including one in the N-terminal regulatory region (Ser 84) and another in the C-terminal regulatory region (Ser 483) (33, 34). This makes PFKFB2 the only PFKFB isoform regulated by multi-site phosphorylation known to date. Indeed, variability between isoenzymes is predominantly in regulatory domains (21). Conversely, the catalytic domains are predominantly conserved (32), demonstrating 76 to 83% sequence homology across isoenzymes and >95% sequence homology amongst human and bovine orthologs of PFKFB2 (35).

**Figure 3:**
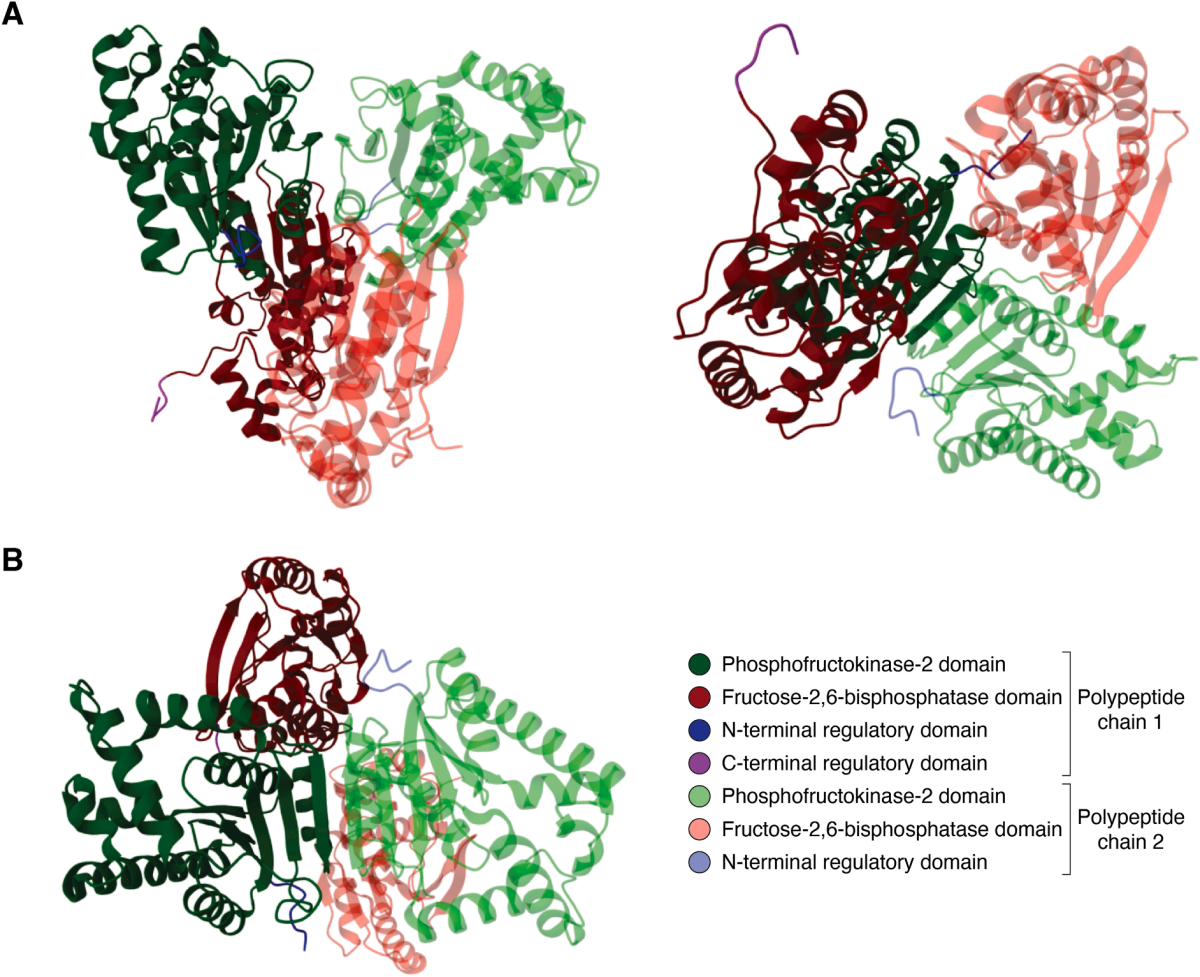
**Molecular structure of PFKFB2.***A*, PFKFB2 is structured as a bifunctional homodimer with both kinase (*green*) and phosphatase (*red*) domains, as well as N-terminal (*blue*) and C-terminal (*purple*) regulatory domains on each polypeptide. *B*, this homodimer is organized in a “head-to-head” conformation due to the interface between kinase domains. Structures were colored using Mol∗(Web GL) from the protein data bank structure 5HTK (https://doi.org/10.2210/pdb5HTK/pdb), deposited in 2016 by Robert B. Crochet. Of note, the structure is truncated at N- and C-terminal regions, such that one C-terminal domain is not shown altogether, presumably due to their failure to crystallize.

In 2017, Crochet *et al.* characterized the conformations of human and bovine PFKFB2 orthologs, supported by crystal structures (35). While properties such as the number of amino acids differed between the orthologs, overall PFKFB2 structure was conserved. Like other PFKFB isoforms, PFKFB2 is a bifunctional homodimer (29, 36). Thus, it is comprised of two identical polypeptides, each containing both a kinase and a phosphatase domain (35) (Fig. 3). The phosphofructokinase domain is localized to the N-terminal region, while the fructose 2,6-bisphosphatase domain is localized to the C-terminal region, with the bifunctional catalytic core flanked by regulatory domains at the termini (35). The polypeptides are oriented in a head-to-head configuration, with dimerization mediated predominantly by kinase domain interaction (35) (Fig. 3). This interface is important for structural stability and enzymatic activity of the kinase domains, whereas the bisphosphatase domains can function independently (29).

### Gene and transcript

The structure, localization, regulation, and splicing of the *PFKFB2* gene were largely established through comparative studies of human, bovine, and rodent models. One distinction amongst PFKFB isoenzymes is their chromosome localization, and this also varies amongst orthologs of the isoenzymes. In rats, *Pfkfb2* is localized to chromosome 13 (37, 38). In humans, the gene encoding PFKFB2 is localized to band 31 of the long arm of chromosome 1 (1q31) (38). Considerably shorter than the *PFKFB1* isoforms, the human *PFKFB2* and rat *Pfkfb2* genes occupy approximately 22 kb (21, 22), though this may vary slightly by species, with the bovine ortholog reported to span approximately 27 kb (24). Across mammalian species, the *PFKFB2* gene displays a highly conserved structural framework, with splicing mechanisms at the 5′ end and coding region serving distinct purposes.

The rat *Pfkfb2* gene utilizes a complex, modular 5′ architecture, where multiple distinct promoters generate at least four mRNAs with alternate 5' regions and at least 3 distinct promoters (R1–R4) (39). Interestingly, at least some of these promoter regions lack a TATA box and that of R4 is GC-rich, implying a reliance on Sp1 binding sites and overlapping transcriptional start sites rather than a single canonical initiation point, while R3 uses an initiator element (Inr) sequence (39). This structure allows for basal expression of different variants across multiple tissues. While the upstream exons differ, they are spliced onto a common exon 2, which contains the primary translation initiation codon (39). This system at the upstream 5′ region allows the decoupling of transcriptional control from the amino acid sequence of the resulting protein, but with the caveat of minicistrons, which encode short peptides implicated in translational control in some mRNAs.

Conversely, diversity in sequence and ultimately protein structure and regulation are generated through alternative splicing mechanisms in the coding region. In parallel with protein-level distinctions, transcripts encoding the catalytic core are relatively conserved (40). Thus, much of the variation induced by alternative splicing exists in 3′ regions encoding the C-terminal regulatory region, which is critical for enzymatic regulation (25). Following the 5' region, and that encoding the N-terminal regulatory segment, exons ∼3 to 7 encode the kinase domain and exons ∼9 to 14 encode the bisphosphatase domain (22, 24). Downstream, exon 15 is specific to the heart isoform of *Pfkfb2*, encodes the Ser 466 and Ser 483 phosphorylation sites, and is a primary site of alternative splicing (24, 25). This splicing results in distinct isoforms with different phosphorylation-dependent regulation (23, 31, 40). In addition to alternative splicing in exon 15, variable presence of different forms of exon 16 may confer variability in regulatory mechanisms and properties (40). This predominantly leads to inclusion of distinct and optional exonic segments that diversify stress response.

## PFKFB(2) catalytic mechanism

**Figure 4:**
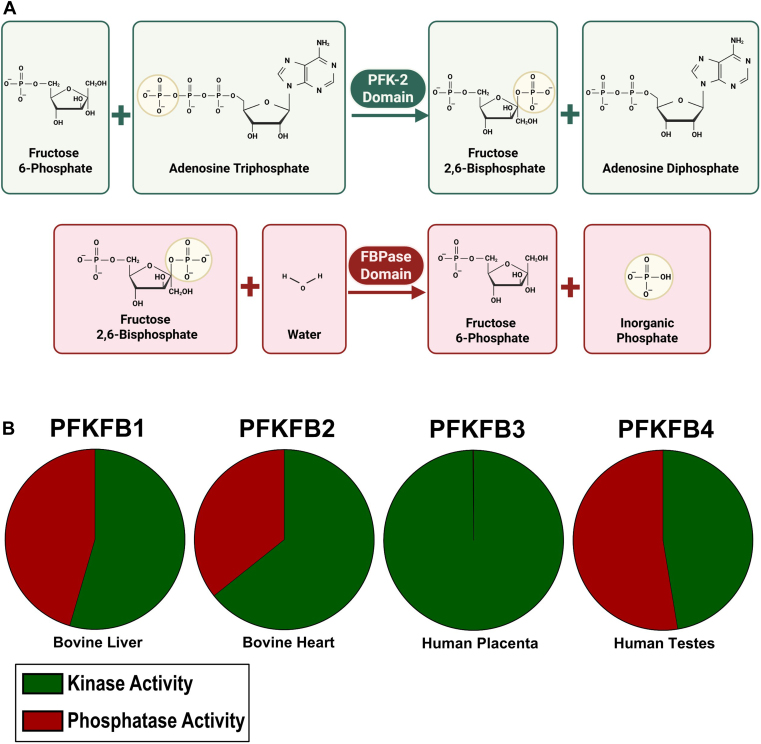
**Catalytic mechanisms and relative activity of PFKFB kinase and phosphatase domains.***A*, reaction mechanism of the Phosphofructokinase-2 domain (PFK-2; *green*) and fructose 2,6 bisphosphatase (FBPase; *red*). The C2 phosphate of fructose 2,6 bisphosphate from pre- and post-transfer and pre- and post-cleavage is shown in *red*. *B*, pie charts depicting relative kinase (*green*) to phosphatase (*red*) activities for each of the different PFKFB isoforms. The tissues from which these measurements were derived is listed directly below the pie chart. Data for panel B were taken from https://doi.org/10.1186/2049-3002-1-8.

As previously mentioned, PFKFB2 and other PFKFB isoforms can catalyze two opposite reactions. The phosphorylation reaction adds a phosphate group to Fru-6-P to produce Fru-2,6-P_2_ with ADP as a remaining byproduct (Fig. 4*A*). The phosphatase reaction is a hydrolysis reaction that cleaves the phosphate from C2 of Fru-2,6-P_2_, yielding Fru-6-P and an inorganic phosphate (Fig. 4*A*). These reactions occur on distinct domains of each polypeptide in the homodimer (35, 36), and are briefly described here. Catalytic reactions are relatively conserved across PFKFB isoenzymes; therefore, this summary of the catalytic mechanism is drawn from knowledge derived from multiple isoenzymes. We highlight key features of these mechanisms here but point to a previous review that spans PFKFB isoforms and highlights the mechanisms in detail (29).

The kinase reaction on the N-terminal half of the polypeptide is an ordered reaction (35) in which ATP binds first, inducing a conformational change that forms a binding pocket for Fru-6-P and aligns catalytic residues (41). Fru-6-P then binds, stabilized by hydrogen bonds, electrostatic interactions, and conformational complementarity, such that the C2 hydroxyl group is in-line with the γ-phosphate of ATP at close proximity (42). This facilitates the OH on C2 attacking the γ-phosphate of ATP without necessitating a phosphoenzyme intermediate (42). The remaining ADP and Fru-2,6-P_2_ then leave and the enzyme returns to the open state.

Conversely, the dephosphorylation of Fru-2,6-P_2_ on the C-terminal half of the polypeptide is a hydrolysis reaction that does proceed *via* a phospho-histidine intermediate. Fru-2,6-P_2_ binds first, and a histidine residue nucleophilically attacks the C2 phosphate (43, 44). A phospho-histidine intermediate then forms and Fru-6-P leaves (43). An H_2_O molecule attacks the phospho-enzyme intermediate, and the inorganic phosphate is released from the histidine (43, 44). Interestingly, experimental data suggest that the phospho-histidine state may be especially stable in PFKFB2 relative to other isoforms (35). The stability of this phospho-histidine intermediate may slow the phosphatase reaction, thereby allowing increased Fru-2,6-P_2_ levels during periods of high metabolic demand. These conserved catalytic motifs highlight the elegant bifunctionality of the PFKFB homodimer, where the spatial separation of domains allows for reciprocal coordination, ensuring a decisive metabolic shift in Fru-2,6-P_2_ levels.

## PFKFB2 regulation

PFKFB2 regulation is complex, arguably even relative to other isoforms. Highlighting this, PFKFB2 is the only isoenzyme known to be multi-site phosphorylated and phosphorylated at both the N- and C-terminal domains (2). This allows it to serve as a true signal integrator, coupling stress, metabolic, and energetic statuses of the cell to glycolytic regulation. PFKFB2’s function as either a kinase or phosphatase based on phosphorylation status allows it to act as a tunable covalent switch. That is, housing both opposing activities on a single bifunctional polypeptide circumvents the futile cycling that opposing monofunctional enzymes may face. Regulation of this mechanism by multisite phosphorylation allows the kinase–phosphatase balance to integrate hormonal, energetic, and stress inputs into a single output, the prevailing Fru-2,6-P_2_ concentration and subsequent glycolytic activity. In this framework, PFKFB2 functions as a single decision node for converting integrated signaling into a glycolytic activity level, rather than as two separate and competing enzymes.

PFKFB2 has a higher kinase:phosphatase activity ratio than PFKFB1 and PFKFB4, but considerably lower than the inducible isoform PFKFB3 which has a kinase:phosphatase ratio >700:1 (2, 45) (Fig. 4*B*). The 1.8:1 kinase:phosphatase ratio of PFKFB2 (2, 45) positions it within range to either upregulate or down-regulate glycolysis dynamically based on cell status. Furthermore, it is important to note that while PFKFB2 is activated by phosphorylation, work from our lab has shown it is degraded when dephosphorylated, further contributing to the nuance of its regulation and suggesting a predominant role in activating glycolysis. Ultimately, while a large focus has been placed on PFKFB2’s post-translational and allosteric regulation, it is regulated at multiple additional levels, including its abundance through epigenetic, transcriptional, and degradation mechanisms.

These regulatory modes operate on distinct timescales and govern distinct aspects of PFKFB2 function. Phosphorylation provides rapid, reversible control over the activity of the existing enzyme pool and dominates acute physiological and stress responses, whereas epigenetic and transcriptional mechanisms set PFKFB2 abundance and basal glycolytic capacity over longer timescales and feature most prominently in chronic disease states such as diabetes and cancer. They are therefore complementary rather than competing, with post-translational control tuning the activity of the protein pool that the slower mechanisms establish.

### Posttranslational regulation

PFKFB2 is phosphorylated at four classically described sites, including three in the C-terminal regulatory domain (Ser 466, Ser 483, and Thr 475) and one (Ser 84) in the kinase domain closer to the N-terminus, and PFKFB2 is typically dephosphorylated by protein phosphatase 2A (PP2A) (33, 46). PFKFB2 can be phosphorylated by protein kinase C (PKC) at Ser 466, Thr 475, and Ser 84; by adenosine monophosphate-activated protein kinase (AMPK) at Ser 466; and by protein kinase B (Akt), protein kinase A (PKA), and p70 ribosomal S6 kinase (p70S6K) at Ser 483 and Ser 466 (25, 33, 34, 47, 48, 49). Of note, the numbering of these phosphorylation sites may vary slightly by species but the seminal studies, conducted primarily in bovine PFKFB2, generally utilize a shared numbering convention with human PFKFB2. Although p70S6K’s role in PFKFB2 phosphorylation is less established, available evidence suggests preferential targeting of Ser 466 over Ser 483 (33). Despite their localization in the domain adjacent to the fructose bisphosphatase domain, phosphorylation at Ser 466 and 483 impacts the kinase domain. Using mutations from serine to glutamate as a phosphomimetic, it was determined that when both Ser 483 and Ser 466 are phosphorylated, the affinity of the PFKFB2 kinase domain for Fru-6-P is increased (50). Additionally, Ser 483 phosphorylation alone alleviates citrate-mediated inhibition and Ser 466 phosphorylation alone is sufficient to increase maximal velocity (Vmax) (50). Biochemical evidence suggests that phosphorylation at Thr 475 by PKCζ may also contribute to activation of PFKFB2, increasing Vmax, though this has been demonstrated *in vitro* and in adipocytes and may not occur in response to insulin in the heart (51). However, while the role of Ser 84 is not well understood, it is thought that it may offset the effects of phosphorylation at the activating sites (2). This adds both nuance and ambiguity to the overall role of PKC, which phosphorylates Ser 84 as well as Ser 466 and Thr 475, suggesting perhaps a self-attenuating mechanism (2).

The Ser 466 and Ser 483 phosphorylation sites are the most studied. As such, the implications of Akt (insulin signaling) and PKA (adrenergic signaling) on the activation of PFKFB2 kinase activity are well established (30, 33, 49, 52, 53, 54, 55, 56). In addition to increasing kinase activity, phosphorylation of PFKFB2 by Akt can also promote protein-protein interactions. When both Ser 466 and Ser 483 are phosphorylated by Akt, 14-3-3 proteins can bind to the phosphorylated Ser 483 residue (57). Disruption of this interaction completely suppresses the insulin-like growth factor-1 (IGF-1)-induced increase in cellular Fru-2,6-P_2_, indicating that 14-3-3 binding is required for glycolytic activation, though the precise mechanism, whether through altered enzyme kinetics or subcellular relocalization, remains to be established (57). This was investigated in the context of IGF signaling but ultimately points to broader mechanisms by which Akt mediates PFKFB2 activation.

The relatively clear understanding of the impact of Ser 483 and Ser 466 phosphorylation helps to delineate the roles of signaling kinases in regulating glycolysis. However, these roles can be contextually nuanced. This is especially true of AMPK-mediated regulation of PFKFB2 at Ser 466. For example, it would be expected that increased workload and subsequent ATP depletion would stimulate AMPK-mediated phosphorylation of PFKFB2 *via* a shift in energy charge. Indeed, the Hue group has shown that with increased workload, PFKFB2 is activated (58). However, under the same conditions, AMPK is not activated (58). This is explained in part by a slight depletion of AMP with no change in ATP levels (58). PFKFB2 is instead activated by a wortmannin-sensitive mechanism when workload is increased, implicating Akt or some other mechanism downstream of PI3K(58). This suggests that in normoxic conditions, at least in the absence of a significant stress beyond increased energetic demand, glycolytic upregulation is AMPK-independent. However, in the instance of ischemia or hypoxia, AMPK activation is sufficient to phosphorylate PFKFB2 (59, 60). Further complicating this paradigm, insulin treatment antagonizes AMPK’s activation in response to ischemia, and ischemia antagonizes insulin signaling (61, 62, 63, 64). Therefore, in the heart, the insulin signaling mechanisms and AMPK activation that result in PFKFB2 phosphorylation exhibit upstream antagonism, despite sharing a common ultimate outcome (65).

### Citrate-mediated regulation of PFKFB2

While phosphorylation predominantly contributes to activation of PFKFB2, citrate-mediated modulation inhibits PFKFB2 by binding to the Fru-6-P binding pocket of the kinase domain. Crystallography demonstrated that citrate also prevents the γ phosphate of an ATP analog from occupying the necessary position for proper catalytic activity, instead forcing an alternate mode of binding (35). Interestingly, kinetic analysis uncovered that citrate led to a non-competitive inhibition mechanism when ATP was varied, consistent with the crystallographic observations that citrate impedes proper ATP binding but does not block it entirely, and that citrate can bind both the free enzyme and the ATP-enzyme complex (35). However, citrate did competitively inhibit Fru-6-P binding, consistent with occupation of its binding site (35). Together, these dual inhibitory interactions provide a molecular basis for citrate-mediated suppression of PFKFB2 catalytic activity. This has important implications for the Randle Cycle, whereby fatty acid oxidation inhibits glucose utilization in part through the accumulation of citrate, which inhibits PFKFB and PFK-1 (66, 67). Interestingly, it has been shown that adrenergic stimulation and phosphorylation of PFKFB2 may be sufficient to overcome citrate-mediated inhibition of PFKFB2 kinase activity (68). In the heart, this has direct physiological relevance, as PKA-mediated phosphorylation of PFKFB2 during adrenergic stimulation may be sufficient to override the Randle Cycle, thereby promoting glycolysis despite the presence of fatty acids.

### Epigenetic

It has also been shown that PFKFB2 transcription can be regulated *via* epigenetic means. In a system where M2 macrophages impact transcriptional regulation of carcinoma cells, PFKFB2 is upregulated (69). When M2 macrophages secreted the chemokine ligand CXCL1, the histone H3 lysine demethylase KDM6B was upregulated in cancer cells (69). This led to demethylation of H3K27me3, a repressive mark, and ultimately promoted PFKFB2 transcription (69). Future studies will be required to determine if PFKFB2 transcription in other tissues is regulated through a similar mechanism.

Methylation of DNA itself also has potential to impact PFKFB2 transcription. Work from Yousri *et al.* used a combination of epigenetic and whole genome sequencing analyses in a cohort of patients with type 2 diabetes to show CpG single nucleotide polymorphisms (CpG-SNPs) in the PFKFB2 gene which predispose it to methylation (70). Furthermore, PFKFB2 methylation was reduced in type 2 diabetes and inversely associated with HbA1c, with Mendelian randomization analysis suggesting that lower PFKFB2 methylation may causally contribute to poorer glycemic control rather than simply reflecting it (70). A recent follow-up further demonstrates a link between PFKFB2 methylation and alterations in triglycerides and lipoproteins within the same Middle Eastern cohort with type 2 diabetes (71). Of note, these studies were performed in a cohort from a population with a high propensity for type 2 diabetes, further affirming the potential role of this epigenetic modulation in propensity or risk of type 2 diabetes.

Finally, an additional study has investigated the role of PFKFB2 promoter methylation in the pathology of thyroid carcinoma (72). This work identified hypomethylation of the PFKFB2 promoter as a key marker of recurrence or relapse in thyroid carcinoma (72). These findings collectively point to epigenetic regulation of PFKFB2 and subsequent implications for metabolic contributions to pathophysiology. This marks an emerging area which necessitates further work across a broader array of physiological conditions.

## PFKFB2 role in cardiometabolic (Patho)physiology

Under baseline healthy conditions, the heart generates 60 to 70% of ATP from fatty acids and 30 to 40% from glucose. However, a central property of cardiac metabolism is metabolic flexibility-the ability to alter the balance of substrate utilization based on either relative substrate availability or other necessities or constraints such as the rate of ATP production or oxygen levels. Under conditions of enhanced sympathetic input or adrenergic stress, when rapid energy production is needed to enhance inotropy, lusitropy, dromotropy, and chronotropy, glucose metabolism may be acutely upregulated. This is supported in large part by PKA-mediated PFKFB2 phosphorylation. As a more baseline response, relative substrate utilization differs in the presence or absence of insulin and, under healthy conditions, concomitant availability of circulating glucose. This is mediated in large part by Akt-mediated phosphorylation of cardiac PFKFB2(52). Interestingly, amino acid supplementation also stimulates Akt to activate PFKFB2, in a manner that is dependent on p38 and PI3K, but insensitive to rapamycin, suggesting an mTORC1-independent mechanism (53). This implicates broader nutrient signaling responsiveness of Akt-mediated PFKFB2 activation (53). Here, we highlight the role of these processes in cardiac (patho)physiology-with focuses on both the protective role of PFKFB2 in hypoxic response, as well as the potential implications of its loss on cardiac pathology in the absence of insulin signaling.

### PFKFB2 loss in cardiac hypoinsulinemia

Two key mechanisms of PFKFB2 activation, insulin signaling and PKA signaling, have an intricate interplay. Specifically, sustained elevation of adrenergic tone impairs insulin signaling and subsequent glucose utilization *via* a PKA-dependent mechanism (73, 74, 75). Reciprocally, cardiac PKA signaling is impaired in the absence of insulin signaling, with PFKFB2 a key substrate desensitized to PKA-mediated phosphorylation with chronic hypoinsulinemia (55, 56). This impaired phosphorylation is likely due, at least partly, to reduced cytosolic localization of catalytic PKA subunits with chronic hypoinsulinemia (56).

In addition to impaired activation of PFKFB2 due to decreased Akt- and PKA-mediated phosphorylation under hypoinsulinemic conditions, PFKFB2 is also degraded with absent or impaired insulin signaling. Four months after treatment with the pancreatic beta cell toxin streptozotocin (STZ), which promotes hypoinsulinemia and hyperglycemia, PFKFB2 protein content was reduced by over 60% in the heart (30). Further, in a high fat diet-induced model of insulin resistance, PFKFB2 protein content was decreased by 20% after 1 week and 25% after 21 weeks, suggesting scalability to degree and duration of insulin resistance (30). On a shorter-term time scale, 24 h of fasting, which produces a relatively acute hypoinsulinemic state, was also sufficient to reduce PFKFB2 levels by approximately 37% (30). Finally, our recent work shows that PFKFB2 is also degraded in patients with heart failure with preserved ejection fraction, a syndrome marked by metabolic disease and particularly insulin resistance (76). Mechanistic studies conducted in isolated primary mouse adult ventricular cardiomyocytes (ACMs) found that insulin, IGF, or PKA signaling were sufficient to sustain PFKFB2 levels (30). This suggests an ultimate relationship between PFKFB2 phosphorylation and stability.

The decreased protein expression in hypoinsulinemia came without a change in *Pfkfb2* transcript levels. Though, it is important to note the aforementioned epigenetic regulation of PFKFB2 in type 2 diabetes which may point to a context specificity of the genetic aspect of regulation (70, 71). That is, epigenetic factors may contribute to PFKFB2 loss in individuals with type 2 diabetes predisposition. However, these factors may not be observed in wildtype preclinical rodent models administered a diabetogenic stress. Nonetheless, there is inevitably a role of PFKFB2 degradation at the protein level in diabetic cardiac pathophysiology.

Further studies in ACMs investigated the mechanisms of PFKFB2 degradation and found it was regulated by two distinct processes. Short-term degradation (4 h time scale) was mediated by proteasomal degradation mechanisms, whereas longer term degradation (24 h time scale) was associated with lysosomal or autophagic mechanisms (30). More specifically, this was attributable to chaperone-mediated autophagy, for which PFKFB2 contains a putative consensus sequence for binding Hsc70 (30). Ultimately, a majority of the degradation which occurs in the absence of insulin signaling is rapid, with cardiac PFKFB2 demonstrating a half-life of 2.2 h in the presence of the translation inhibitor cycloheximide and the PI3K inhibitor wortmannin (30). While perhaps counterintuitive given the critical importance of PFKFB2 in glycolytic regulation, some PFKFB2 degradation may be protective given the bifunctionality of PFKFB2; *that is*, if dephosphorylated PFKFB2 was stable, its phosphatase activity may excessively deplete the Fru-2,6-P_2_ pool. However, while there may be physiological cause for this degradation under healthy conditions, chronic degradation inevitably impacts cardiac metabolic programming and flexibility.

To assess the implications of this PFKFB2 depletion, we generated a cardiomyocyte-specific knockout model. Our goal was to determine how the loss of PFKFB2 affected cardiac metabolism and function without the confounding effects of hypoinsulinemia and cardiometabolic stress. Indeed, PFKFB2 loss did suppress metabolic flexibility (77) and promoted broad metabolic reprogramming, with ancillary glucose pathway activation amongst the most prominent features (78). One example of this is upregulation of enzymes involved with both the oxidative and nonoxidative branches of the pentose phosphate pathway in mouse PFKFB2 knockout hearts (78). This is in alignment with data showing a buildup of glycolytic intermediates upstream of the phosphofructokinase nexus and intermediates of the pentose phosphate and glycogen synthesis pathways, but downregulation of glycolytic intermediates downstream of the phosphofructokinase nexus, following 24 h of fasting in wildtype mice (30). These data collectively point to a buildup and subsequent spillover of glycolytic intermediates upstream of the phosphofructokinase nexus when it is impaired with loss of PFKFB2. This is consistent with kinase dominance of PFKFB2, as previous work has shown enhanced flux into ancillary pathways when Fru-2,6-P_2_ is depleted (79).

Consistent with diversion of glucose into ancillary pathways, protein O-GlcNAcylation, a result of the ancillary hexosamine biosynthesis pathway that is increased in the diabetic heart (80, 81, 82, 83, 84, 85), was also enhanced in PFKFB2 knockout hearts (78). Though, the increase in protein O-GlcNAcylation was ameliorated by fasting (78). Conversely, increased cardiac Fru-2,6-P_2_ levels are sufficient to decrease levels of UDP-GlcNAc, the product of the hexosamine biosynthesis pathway and the direct precursor for O-GlcNAcylation, in a high fat diet-induced model of insulin resistance (86). Collectively, these data support coordination between substrate availability and the phosphofructokinase nexus, undeniably regulated by PFKFB2 levels and activity (77).

Beyond promotion of increased O-GlcNAcylation levels, PFKFB2 loss also drives many of the pathological phenotypes which occur with more direct O-GlcNAc upregulation. These phenotypic similarities include cardiac dilation, ventricular arrhythmogenesis, and sudden death propensity (76, 78, 87). Interestingly, however, the duration of ventricular arrhythmogenesis was protected by the same fasting duration that ameliorated the increase in O-GlcNAcylation in PFKFB2 knockout hearts (76, 77). These data support a critical role of PFKFB2 loss in promoting the pathology of metabolic heart disease *via* O-GlcNAcylation.

**Figure 5:**
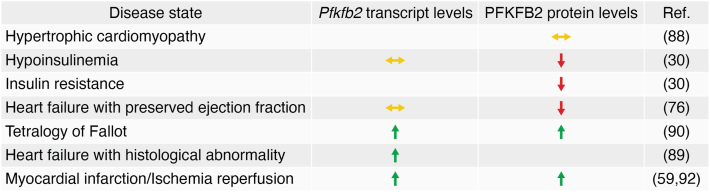
**Changes in cardiac PFKFB2 abundance in cardiac pathology.** Protein and transcript levels of cardiac PFKFB2 are shown across disease states. Downward *red arrow* indicates a decrease in disease, upward *green arrow* indicates increase in disease, and sideways *yellow arrow* indicates no significant change in PFKFB2 in the disease.

However, while PFKFB2 loss is observed in metabolically associated pathologies such as heart failure with preserved ejection fraction (76), as well as diabetic models of both insulin resistance (induced by high fat diet) and hypoinsulinemia (induced by streptozotocin), PFKFB2 is sustained in hypertrophic cardiomyopathy (88) (Fig. 5). This is consistent with the notion that PFKFB2 degradation is specific to metabolic diseases, and particularly those marked by impaired insulin signaling. Reciprocally, a spatial transcriptomic study found that *PFKFB2* expression is elevated in histologically normal myocardium from compensated cardiomyopathy patients, but relatively reduced in uncompensated end-stage, structurally abnormal myocardium (89). This pattern suggests that PFKFB2 may be associated with myocardial compensation in heart failure that arises in the absence of overt systemic metabolic derangements, although causality remains untested. Supporting a broader role for *PFKFB2* variation in human cardiac pathology, rare-variant analysis in the UK Biobank cohort identified loss of function (LoF) and missense *PFKFB2* variants as associated with heart failure and cardiomyopathy (89). Interestingly, population-level constraint analysis indicates that *PFKFB2* is tolerant to heterozygous LoF, reflected by a pLI (probability of loss-of-function intolerance) score of 0, yet exhibits moderate missense constraint (Z = 2.95). This supports that a subset of missense variants may impair protein function despite heterozygous LoF being generally well tolerated (89). The absence of an identified Mendelian disorder associated with biallelic *PFKFB2* variants may reflect an essential developmental or cardiac role rendering homozygous loss incompatible with viability. Collectively, these findings support the conclusion that dysregulation or deleterious variations in *PFKFB2* is linked to heart failure pathology in humans, though establishing causality will require deeper functional investigation.

### PFKFB2 in cardiac hypoxic response

Despite the unchanged PFKFB2 in adult hearts with hypertrophic cardiomyopathy, right ventricles of infants with the hypoxic congenital heart defect Tetralogy of Fallot exhibited increases in both PFKFB2 transcript and protein abundance (90). This suggests that while changes in PFKFB2 may not accompany structural heart disease alone, there could be a greater propensity for PFKFB2 upregulation when structural heart disease promotes or is accompanied by hypoxia. Indeed, PFKFB2 is transcriptionally upregulated in multiple tissues or cell lines in response to hypoxia or a host of hypoxia mimetics including hypoxia-inducible factor (HIF) hydrolase inhibitors, iron chelators, and transition metals (91). Animal exposure to environmental hypoxia was, itself, insufficient to upregulate PFKFB2 in the heart (91). However, the magnitude of hypoxia conferred by environmental exposure may be insufficient to drive the upregulation, as studies have shown upregulation of PFKFB2 transcript and protein levels with more direct models of myocardial infarction (92) and ischemia/reperfusion (59). Beyond abundance, myocardial infarction also leads to enhanced PFKFB2 phosphorylation and thus activation, suggesting that PFKFB2 activity is enhanced by multiple mechanisms in response to cardiac ischemia.

PFKFB2 upregulation and activation is an unsurprising adaptive mechanism that accompanies a host of complementary processes to upregulate cytosolic glucose metabolism in response to hypoxia and HIF stabilization in the heart (93). This allows for metabolic compensation *via* anaerobic processes such as glycolysis when aerobic metabolism is compromised due to impaired tissue oxygen delivery. Indeed, PFKFB2 overexpression confers cardioprotection through amelioration of apoptosis in response to myocardial infarction (92) and amelioration of ferroptosis in response to oxygen/glucose deprivation/reperfusion in H9C2 cells and ischemia/reperfusion *in vivo* (59). While the former was attributed largely to a PFKFB2-mediated increase in glycolytic flux (92), the latter occurred with glucose deprivation and implicates PFKFB2-mediated AMPK activation (59). Though, in support of glycolytic upregulation itself being sufficient to promote cardioprotection, overexpression of a phosphatase-deficient PFKFB1, which upregulates Fru-2,6-P_2_ levels, protects primary isolated cardiomyocytes against hypoxia-mediated contractile impairment (94). Furthermore, the reciprocal model, overexpression of a kinase-deficient PFKFB1 that promotes Fru-2,6-P_2_ deficiency, exhibits impaired function under hypoxic conditions (1).

PFKFB2 activation is amongst multiple shared glucose metabolism targets driven by both insulin and ischemia through distinct pathways, a phenomenon previously analyzed and reviewed by Hue *et al.* (65). Interestingly, vascular rarefaction can drive hypoxia and ischemic regions in the diabetic heart (95); yet PFKFB2 is nonetheless degraded and glucose metabolism suppressed. Under ischemic conditions such as infarct alone, hypoxia-mediated HIF activation promotes Akt activation and subsequent PFKFB2 phosphorylation. However, HIF stabilization is impaired in the myocardium under hyperglycemic or diabetic conditions (96, 97, 98), as previously reviewed in detail (99). This not only contributes to metabolic dysfunction in the heart in diabetes but highlights a relationship between insulin signaling and hypoxia whereby the loss of PFKFB2 prevents protective responses.

## PFKFB2 across noncardiac tissues and systems

While initially purified from the heart and known in the literature as the “cardiac isoform”, PFKFB2 transcript is expressed in various tissues. Even in tissues which predominantly express a different isoenzyme, PFKFB2 may play important roles due to its capacity for signal integration. For example, skeletal muscle predominantly expresses PFKFB1, but it has been demonstrated that PFKFB2 plays a role in the tissue’s insulin response (100). Similarly, PFKFB3 is implicated in adipose tissue metabolic regulation, but the therapeutic agent Salvianolic acid B has been shown to act on adipocytes to confer greater glycolytic capacity in a process associated with upregulation of PFKFB2 (101). Indeed, the human protein atlas identifies high expression in non-cardiac tissues such as retina, thyroid gland, and skeletal muscle (Fig. 2). However, other data sets such as those on Bio GPS show considerably higher gene expression in the heart than other tissues. Given the differences in splicing amongst isoforms localized to distinct tissues, it is plausible that these differences may arise from differential variant recognition by different probes used in detection. Regardless, understanding the role of PFKFB2 across tissue types is important, especially in understanding potential implications of systemic modulation.

### Eye

Despite high retinal PFKFB2 expression, specifically in metabolic gatekeeping layers such as the retinal pigment epithelium (RPE), limited literature exists investigating the role of PFKFB2 in the eye. In Huntington’s disease, PFKFB2 is transcriptionally downregulated, the implications of which are unknown (102). Conversely, in retinoblastoma, PFKFB2 is upregulated, and its suppression blunts tumorigenesis (103). This likely only scratches the surface of PFKFB2’s role in ocular pathophysiology. For example, given the high retinal PFKFB2 expression (Fig. 2) and the degradation of cardiac PFKFB2 with impaired insulin signaling (30), an interesting question concerns the levels, activity, and subsequent implications of PFKFB2 in diabetic retinopathy, as well as how locally produced insulin impacts this modulation (104). For example, if the RPE pool of PFKFB2 is similarly depleted in diabetes or unresponsive to locally produced insulin, the resulting loss of metabolic flexibility could disrupt the nutrient support provided to the photoreceptors. The discrepancy between high retinal PFKFB2 expression and limited associated literature marks a necessary area for future investigation.

### Liver

The presence of PFKFB2 in the liver has fascinating implications given its distinct and often reciprocal regulation from the liver PFKFB1 (L-PFKFB1), the predominant hepatic isoenzyme. In the liver, the role of PFKFB and Fru-2,6-P_2_ is to regulate both glycolysis and gluconeogenesis. Additionally, while tissues such as the heart upregulate glycolysis in response to PKA signaling, the reciprocal is true of the liver which mobilizes glucose for circulation (2). Therefore, glucagon-mediated gluconeogenesis signals through PKA and inhibits L-PFKFB1 kinase activity. To facilitate this, PKA phosphorylates the N-terminus of L-PFKFB1 which inhibits kinase activity and promotes phosphatase activity. Thus, the impacts of hepatic PKA activation on PFKFB1 decrease Fru-2,6-P_2_ pools, inhibit glycolysis, and promote gluconeogenesis (2, 105, 106), in contrast to the effects of PKA on PFKFB2. Interestingly, insulin attenuates the effects of glucagon, cAMP, and epinephrine on the Fru-2,6-P_2_ pool in the liver (107). This supports the possibility that PFKFB2, whose kinase domain is activated by intracellular signals downstream of each of these factors, may counter the effects of L-PFKFB1 on the Fru-2,6-P_2_ pool. A physiological context of relevance is cold stress, where hepatic sympathetic stimulation is augmented, activating gluconeogenesis (108, 109) in line with enhanced PFKFB1 phosphatase activity. However, several studies have shown that PFKFB2 phosphorylation, a surrogate marker of activity, is also enhanced with cold stress, dependent on the duration of stress (110, 111, 112). This interestingly highlights a setting where L-PFKFB1 and PFKFB2 exert functionally opposing roles in the liver, a seemingly contradictory relationship that may be distinguishable by functional nuances, such as temporal differences.

While the role of PFKFB2 in the liver is seemingly complex, pathophysiology highlights the importance of hepatic PFKFB2. For example, PFKFB2 has been implicated in epithelial-mesenchymal transition of hepatic stellate cells by driving aerobic glycolysis, which facilitates STAT3 lactylation and subsequent nuclear translocation (113). Conversely, heightened PFKFB2 activity enhances liver regeneration through glycolytic upregulation (46). This highlights the context-dependent roles of PFKFB2 in the liver, as well as its interplay with L-PFKFB1, as prime areas for future investigation.

### Kidney

Among the first models of genetic PFKFB2 manipulation were transgenic mice with a knock-in of PFKFB2, whereby Ser468 (Ser 466) and Ser485 (Ser 483) phosphorylation sites were mutated to alanine residues (114). The Power lab, who generated this model, focused on its effects in the kidney based on the precedent that glycolytic regulation differed by kidney region and impacted renal fibrosis (114). They found that the transgenic mice exhibited increased renal fibrosis, particularly in the unilateral ureteric obstruction model. The predominant effects were suggested to likely be in the distal renal tubules where glycolytic activity is higher (114). In a follow-up study, the Power group tested whether the established anti-fibrotic effects of metformin acted through PFKFB2 activation. They treated wildtype and PFKFB2 transgenic mice with metformin in the unilateral ureteric obstruction model. This approach follows logically as PFKFB2 is an AMPK substrate. However, the effects of metformin on fibrosis were uniform across wildtype and transgenic groups, suggesting independent antifibrotic mechanisms (115). Though, this does not rule out a potential role of PFKFB2 therapeutic targeting for renal fibrosis and related disease. Indeed, an integrative proteomic and transcriptomic study identified PFKFB2 as a potential therapeutic target for chronic kidney disease and improvement in renal function, supported by consistent directionality of associations between PFKFB2 expression or protein abundance and kidney function (116).

The positive correlation between PFKFB2 and renal function extends to additional disease conditions. For example, genetic variants of PFKFB2 that result in decreased PFKFB2 expression conferred susceptibility to diabetic nephropathy in a Pima Native American population (117). This association may be supported by specific upstream regulators, such as XIST, which sequesters miR-122-5p (a liver-enriched microRNA that primarily regulates hepatocyte proliferation, differentiation, and lipid metabolism) to decrease inhibition of PFKFB2 expression in response to renal stress, as previously noted in acute kidney injury following transplantation (118). Interestingly, PFKFB2 and other PFKFB isoenzymes are elevated in urinary extracellular vesicles following kidney transplantation in cases of T cell-mediated rejection (119). While causality is difficult to establish in this study in the absence of chronological clarity, this may represent a protective metabolic response to mechanisms of T cell infiltration and rejection in the kidney. Finally, PFKFB2 is upregulated in patients with augmented renal clearance (120). Although identified in the context of pathological hyperfiltration, this finding nonetheless describes a scenario in which PFKFB2 expression correlates with increased glomerular filtration. If this relationship proves to be causative, PFKFB2 may represent a target that could be harnessed when renal function is impaired.

### Reproductive system

PFKFB2 has also been implicated in placental and pre-eclamptic pathophysiology. PFKFB2 is upregulated in pre-eclampsia, a change which impacts both cell proliferation and apoptosis in trophoblast cells of the placenta (121). This is not limited to the placenta, as urinary exosomes also show enhanced PFKFB2 abundance and phosphorylation with pre-eclampsia, suggesting increased abundance and activation in the kidney as well (122). Beyond maternal health, the role of PFKFB2 in reproduction extends to impacts on embryonic and fetal development. Interestingly, placental upregulation of PFKFB2 is also observed in intrauterine growth restriction (IUGR) and is associated with fetal cord blood hypoglycemia (123). This interestingly points to increased placental PFKFB2 abundance as a unifying factor between IUGR and pre-eclampsia, a frequent co-morbidity. This suggests that glycolytic activation by PFKFB2 is not only associated with maternal pathology but partitions glucose away from the fetus, which is metabolically dependent on maternal supply for development. Finally, PFKFB2 is also upregulated in endometria of heifers whose embryos arrested at an early stage of development relative to those with viable embryos (124). Collectively, current studies regarding female reproductive health suggest associations between maternal PFKFB2 upregulation and adverse reproductive outcomes.

## PFKFB2 in disease and stress response

As a signal integrator, PFKFB2 is highly implicated in an array of stress responses such as response to exercise or hypoxia. However, due to its robust metabolic implications, dysfunction of the PFKFB2 nexus also has vast implications for pathophysiology. In cases such as diabetes (30, 78) or intervertebral disk degeneration (125, 126), loss of PFKFB2 and subsequent metabolic impairment can contribute to pathophysiology. Contrarily, in the case of cancer cells, PFKFB2 can be hijacked to support the energy demands of pathological proliferation.

### Diabetes

In our discussion of cardiac pathophysiology, we described the implications of PFKFB2 loss on the diabetic heart. However, changes in PFKFB2 levels are also likely involved with the genesis of diabetes itself and the pathological implications of diabetes in other non-cardiac tissues. The former is due to direct roles for PFKFB2 in pancreatic cellular proliferation (127) and function (128), with PFKFB2 upregulation also observed in the differentiation of insulin-producing cells (129). PFKFB2 is expressed in pancreatic islets of various species and plays a critical role in glucose-induced insulin secretion (128). Interestingly, the role of PFKFB2 in this property of pancreatic β-cells seems to be mediated by the protein itself, independent of Fru-2,6-P_2_ levels (128). Furthermore, PFKFB2 expression is decreased in pancreatic islet cells from human cadavers with type 2 diabetes relative to non-diabetic islets (130). Differentially expressed gene network connectivity also suggested a role of PFKFB2 as a part of a glucose response module that is disrupted in diabetic islets (130). This may implicate PFKFB2 loss in impaired glucose-induced insulin secretion in diabetes; though, directionality cannot be established from gene expression levels alone, necessitating deeper investigation into the implications of pancreatic PFKFB2 loss in diabetic pathogenesis and progression.

Beyond the roles of PFKFB2 in pancreatic endocrine functionality, it may impact exocrine function as well. Indeed, pancreatitis is often comorbid with diabetes, an association thought to be due to pathological implications of impaired local insulin secretion and subsequent impacts on acinar cells (131). PFKFB2 may gate this relationship by linking local insulin signaling to acinar cell energy production, facilitating enhanced Ca^2+^ pump activity, and preventing pathological cytosolic Ca^2+^ overload (131). This highlights PFKFB2 activation as a therapeutic target for acute pancreatitis. Further, this draws an interesting parallel with cardiac pathophysiology, where glycolytic ATP production is preferentially utilized for Ca^2+^ and other ion handling (132, 133, 134), a relationship likely impacted by insulin signaling and PFKFB2 activation.

Limited studies in tissues besides the heart and pancreas demonstrate variation in PFKFB2 levels, splicing, and epigenetic regulation with diabetic pathology. For example, in a rat model of diabetes, PFKFB2 expression is decreased in the lung and kidney (135). Additionally, there are multiple splice variants observed in the lung and kidney with diabetes, including three which are kinase-null and would ostensibly further suppress the Fru-2,6-P_2_ pool and impair glycolysis (135). This is in addition to aforementioned work demonstrating body mass index-associated genetic variation in PFKFB2, which is correlated with decreased PFKFB2 levels in the kidney and propensity for diabetic nephropathy (117). Further, multiple identified epigenetic changes associated with PFKFB2 are observed in diabetes (70, 136). Therefore, it is likely that changes in PFKFB2 are implicated in diabetic pathophysiology in numerous additional tissues, another promising area for future work.

### Hypoxia, proliferation, and cancer

As also previously discussed in the context of cardiac pathophysiology, PFKFB2 plays a crucial role in many tissues’ response to hypoxia, allowing them to upregulate anaerobic glycolysis and prevent cell death. Most of this work has focused on cardiac physiology (59, 92) and cancer metabolism (137, 138). Central to the metabolism of cancer is glycolytic regulation in a principle referred to as the Warburg effect (139). This phenomenon, identified by Otto Warburg in the 1920s, describes that cancer cells utilize glycolysis and fermentation of pyruvate to lactate, regardless of tissue oxygen levels (140, 141). This metabolic approach exchanges energetic efficiency for temporal efficiency, allowing rapid energy production to sustain growth and proliferation. Depending on the tumor’s oxygen tension, the Pasteur effect may also come into play, where hypoxia further promotes glycolytic preference. A preponderance of studies on the effect of PFKFB in cancer metabolism have focused on the inducible isoform PFKFB3, particularly given its considerable kinase predominance, which favors glycolytic activation (142). However, there are also identified roles of PFKFB2 in cancer metabolism, with PFKFB2 often contributing both to the Warburg effect and to hypoxia-induced glycolytic regulation in cancer cells (91, 138, 143, 144, 145, 146, 147).

PFKFB2’s signal integration capacity through post-translational and genetic regulation makes it stand out as a context-dependent regulator of cancer metabolism, linking glycolytic control to extracellular signaling and cellular state. By modulating levels of Fru-2,6-P_2_, PFKFB2 fine-tunes glycolytic flux, positioning it as a dynamic contributor to metabolic plasticity in tumors, as well as serving a nodal role in related processes such as biosynthesis (137, 148). While this generally leads to glycolytic upregulation (145, 149, 150), the flexibility is in contrast to PFKFB3, which generally constitutively promotes glycolysis.

A strong focus in the literature at present is on the role, regulation, and modulation of PFKFB2 in gastrointestinal cancers (143, 151, 152, 153, 154, 155, 156, 157), osteosarcoma (138, 144, 158, 159, 160), glioma (137, 161, 162, 163, 164), and female reproductive cancers (69, 149, 165, 166, 167). Interestingly, these predominantly present as solid tumors, an architecture which may favor a hypoxic microenvironment. In addition to its role in metabolic flux control, PFKFB2-regulated glycolysis has been linked to downstream effects on proliferation and survival (127, 152, 161, 168, 169). Though, the magnitude and direction of these effects appear highly context-dependent (170, 171, 172), with few studies even showing PFKFB2 underexpression (173) in cancerous tissue or association with better outcomes (159). Indeed, the nuanced role of PFKFB2 in flux regulation is implicated not only in glycolytic control, but also in broader metabolic partitioning.

Beyond transcriptional regulation of PFKFB2 expression level (147, 161, 174), considerable regulation of PFKFB2 by microRNAs in cancer (155, 156, 160, 163, 164, 167) can also influence its effects on tumor characteristics and metabolism. Furthermore, there is a bidirectional relationship between PFKFB2 and epigenetics. Epigenetic changes influence metabolism *via* PFKFB2 regulation (69, 72, 158), while PFKFB2 increases tumor burden through epigenetic modulation *via* histone lactylation (154). Collectively, these findings support a model in which PFKFB2 functions as an integrative metabolic node in cancer, coupling signal-responsive glycolytic regulation to cellular programs that extend beyond energy production alone (154). This positions PFKFB2 to impact not only pathogenesis but also drug sensitivity (166, 175), metastasis (149, 152), and relapse risk (72, 153). Thus, PFKFB2 may serve as a diagnostic or prognostic biomarker, either alone or in complex with a signature of other markers (153, 176, 177). Though, the complexity and nuance of the relationship between PFKFB2 and cancer, coupled with the potential cardiotoxicity of PFKFB2 inhibition, suggests that other isoforms such as PFKFB3 may serve as better targets for cancer therapy. We point readers to previous reviews which highlight the roles of PFKFB2 and other PFKFBs in cancer metabolism in greater depth (2, 178, 179).

Implications of PFKFB2’s proliferative support are not limited to cancer. Excessive proliferation of pulmonary arterial smooth muscle cells is a hallmark of pulmonary hypertension, contributing to pathological narrowing of the pulmonary artery lumen (180). This proliferative phenotype is tightly linked to hypoxia, which drives a metabolic shift toward glycolysis (180). PFKFB2 expression is upregulated as part of the hypoxia-induced glycolytic program-a response triggered, at least in part, by mitochondrial fission in this context (180). This suggests that PFKFB2 supports the metabolic reprogramming required for pulmonary arterial smooth muscle cell survival and proliferation under low-oxygen conditions, positioning it as a potential contributor to hypoxia-driven vascular remodeling.

### Immune/sepsis

The role of PFKFB2 in immune cell activation is likely involved in some capacity with many of the other pathologies described in this review. For example, how immune cells contribute to the pathogenesis of cancer and infiltrate tumors is a key area of study, and one in which PFKFB2 is likely involved (138, 181). Another example is myocardial infarction, in which lower PFKFB2 expression is a marker of the transition from “acute” to “old” myocardial infarction in peripheral blood, a signature likely due largely to PFKFB2 expression in mononuclear cells (182). Indeed, current literature supports that PFKFB2 is implicated in predominantly mononuclear cells, across both the innate and adaptive immune systems.

Among adaptive mononuclear cells, T cells express PFKFB2 in addition to other PFKFB isoenzymes. PFKFB kinase activity is critical for T cell activation, though this was previously studied in the context of preferential PFKFB3 inhibition (183). However, a PFKFB2-specific contribution is supported through gene set enrichment analysis. This demonstrated that PFKFB2 is part of a lactylation-related 6 gene signature associated with T cell activation (184). Furthermore, the chemokine CCL5 can induce PFKFB2 activation in an AMPK-dependent manner in activated T cells, suggesting that PFKFB2 contributes to metabolic support of activated T cells in chemotaxis (185). Among innate mononuclear cells, dendritic cell maturation can be supported by PFKFB2 activation (186). Additionally, emerging evidence shows that activation of PFKFB2 is implicated in a broader PIM2-mediated metabolic reprogramming toward aerobic glycolysis that supports macrophage M1-like polarization and inflammation in arthritis (187).

Interestingly, another recent study has shown that glycolytic upregulation and lactate production secondary to PFKFB2 activation are also both implicated in promotion of efferocytosis (188). Moreover, PFKFB2 is itself activated by efferocytosis *via* an Akt-dependent mechanism, and this role is seemingly specific to resolving-type rather than pro-inflammatory macrophages (188). This suggests that PFKFB2-mediated regulation of glycolysis is not only essential for efferocytotic feedback, but also represents a critical context-dependent nexus. Specifically, it regulates feedback between macrophage polarization and efferocytosis, distinguishing pro-inflammatory and pro-resolving pathways (188). This role of PFKFB2 in efferocytotic-specific glycolysis, in contrast to PFKFB3 which is associated with classical inflammatory glycolysis, highlights an important conceptual advance in our understanding of the relationship between glucose metabolism and inflammatory mediation.

While a large proportion of the literature on PFKFB2 in immune response has focused on mononuclear cells, PFKFB2 is also implicated in other cell types and pathologies such as endotoxemia, sepsis, and lung injury. Indeed, under endotoxemic conditions, PFKFB2 is upregulated in neutrophils to fuel their response to endotoxin *via* glycolytic energy production (189). More broadly, evidence suggests that the gut microbial metabolite indole-3-lactic acid binds stably to PFKFB2. This suggests that PFKFB2 may mediate part of the gut microbial influence on host response in sepsis, though the direct implications, cell type specificity, and role of this interaction in both sepsis and potential healthy conditions remain to be elucidated (190). Furthermore, PFKFB2 also contributes to septic lung injury under endotoxemic conditions, where its promotion of glycolysis in fibroblasts contributes to a pro-inflammatory and pro-fibrotic milieu (191). This is likely only one mechanism of PFKFB2-mediated lung injury, as PFKFB2 plays an important role in dendritic cell activation and inflammation, worsening lung injury across multiple diseases (186). Paradoxically, in alveolar regeneration, PFKFB2 plays a crucial protective role in metabolic support of alveolar type 2 (AT2) cell differentiation (192). This makes PFKFB2 therapeutic targeting in this context nuanced, both regarding temporal- and cell type-specificity. Finally, *Pfkfb2*’s position directly upstream of regulators of complement activation is remarkably conserved across species and thus consistently used as a genomic landmark (193, 194, 195). This raises questions surrounding a potential functional basis for this positional conservation, and whether PFKFB2 may be involved in additional aspects of immunological response.

## Conclusion and future directions

**Figure 6:**
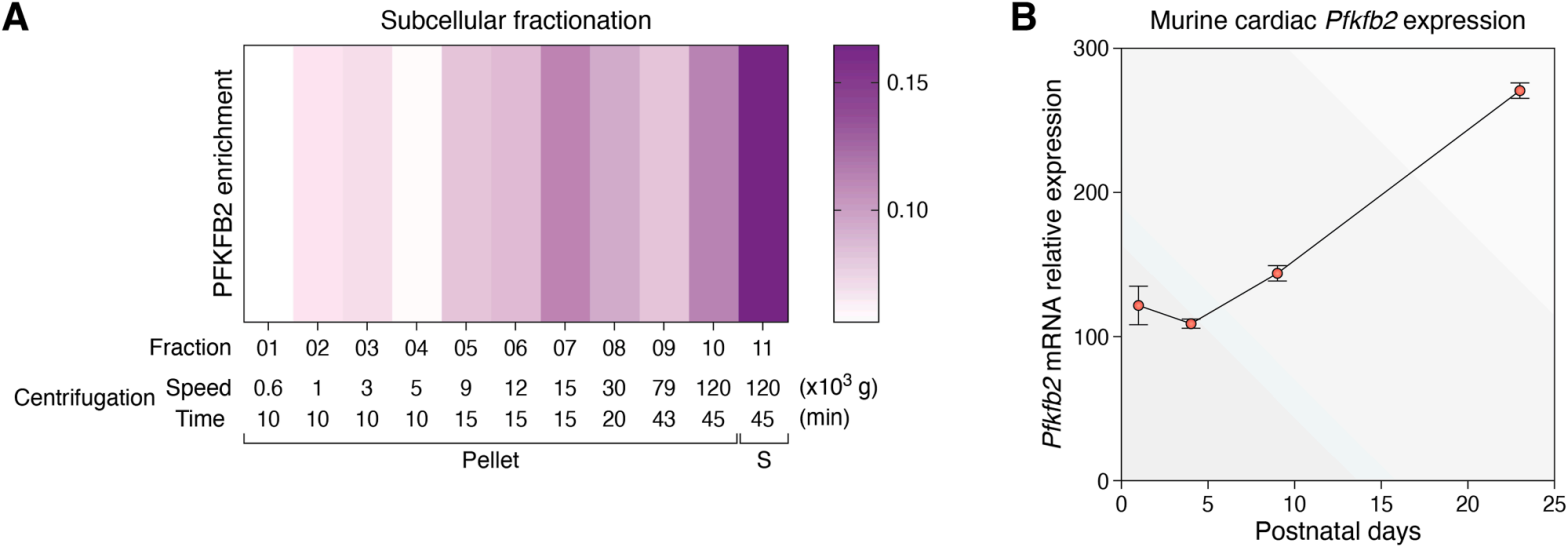
**PFKFB2 implications for future directions.***A*, PFKFB2 is highly enriched in the cytosolic fraction as demonstrated by a differential centrifugation experiment with PFKFB2 levels by fraction in *purple* (*top*) and the corresponding centrifugation speed and time indicated below. Fractions 1 to 10 represent resulting pellets, whereas the 11th fraction is the final supernatant (S). This panel was derived from data found in a study published at https://doi.org/10.1016/j.mcpro.2025.100952. *B*, Pfkfb2 mRNA expression in early postnatal development is increased from days 5 to 25. Data taken from a study published at https://doi.org/10.1161/JAHA.118.010378.

While originally identified in the heart, PFKFB2 has since been shown to be involved with physiology and pathophysiology across other cells, tissues, and systems. Undeniably, PFKFB2 is understudied, and there is much left to be discovered regarding the roles and regulation of PFKFB2 in health and disease. For example, few PFKFB2 variations have been observed across ethnic populations, suggesting room for investigation into the role of *PFKFB2* variation in health disparities (196). Another interesting potential area of investigation is the role of PFKFB2 or Fru-2,6-P_2_ in processes unrelated to glycolysis and glucose use regulation. PFKFB3 can translocate to the nucleus and has been implicated in processes such as DNA repair *via* local Fru-2,6-P_2_ production (197). While PFKFB2 seems to be predominantly cytosolically localized (Fig. 6*A*) (198), it remains to be determined whether it has additional functional roles in the nucleus, similar to those of PFKFB3.

Another underinvestigated area is the role of PFKFB2 in development. The fetal heart relies predominantly on glycolysis and shifts toward fatty acid oxidation throughout development. However, a counterintuitive increase in PFKFB2 transcript expression is observed through development (Fig. 6*B*) (199). One possibility is that increased PFKFB2 levels throughout development support an increased need for metabolic flexibility, and particularly flexibility in glucose utilization, as fatty acid oxidation increases.

Finally, a promising area of ongoing work building on the principles described in this review focuses on therapeutic modulation of PFKFB2. This itself is nuanced, with PFKFB2 inhibition generally protective in pathologies such as cancer, and PFKFB2 activation more protective in many other pathologies. As alluded to, inflammation is more complex and nuanced with the directionality of beneficial PFKFB2 modulation being context-dependent. Small molecules, such as the recently identified B2, have potential to preferentially inhibit PFKFB2 kinase activity (200). Additionally, cancer therapeutics have included traditional plant-derived compounds such as Shikonin, Hesperidin, and Triptolide which downregulate PFKFB2 and inhibit the Warburg effect amongst other tumorigenic and invasive properties in cancer (157, 201, 202). However, as informed by implications of PFKFB2 loss in tissues such as the heart, pancreas, and kidney in other diseases, these cancer therapeutics may have deleterious impacts on physiology in other tissues. This should be considered when pursuing this method of cancer metabolic modulation.

Conversely, while elevation of Fru-2,6-P_2_ levels *via* phosphatase-null PFKFB1 overexpression confers protection in cardiac disease (86, 203), pharmacological activation of PFKFB2 itself has proven rather elusive. This is limited by the lack of PFKFB2 isoenzyme-specific activators. While PFKFB2 is expressed in several tissues, as highlighted in this review, PFKFB2 has the highest relative cardiac expression to other isoenzymes, and therefore isoenzyme-specific modulators should be used to optimize efficacy with minimal off-target implications. For instance, one could speculate that a broad-spectrum PFK activator which also activates isoenzymes such as PFKFB3 may support cancer progression; though presumably, a carcinogenic event would serve as a prerequisite for such a concern. On the contrary, as described herein, current knowledge supports the notion that PFKFB2 activation may confer protection in several other tissue types, such as the kidney and pancreas, especially under conditions such as hypoxia or diabetes. Nonetheless, therapeutic priorities in diabetic and cardiac metabolic pathologies should focus on both isoenzyme specificity and tissue-specific delivery. A study in aged mice reinforced confidence in the potential safety of this approach once specificity is achieved, in that increased Fru-2,6-P_2_ levels in the heart across the entirety of the murine lifespan had limited adverse effects on cardiac pathophysiology (204).

Taken together, the literature reviewed here demonstrates how PFKFB2 serves as a context-dependent metabolic regulator whose regulation itself and functional impacts are determined by an array of integrated signals. Across cardiac, renal, immune, hepatic, and several other systems, PFKFB2 emerges as a response node for a wide range of different stress or energetic changes. Key future priorities include defining tissue- and cell-specific regulatory mechanisms, resolving whether PFKFB2 participates in non-canonical functions beyond glycolytic control, and clarifying how developmental and evolutionary pressures have shaped its conserved genomic and expression patterns. From a translational perspective, the dualistic roles of PFKFB2 under both health and disease conditions emphasize the need for precision approaches that combine isoenzyme specificity with spatial and temporal targeting. Addressing these gaps will be essential not only for understanding PFKFB2’s metabolic and potential non-metabolic roles, but also for determining whether selective modulation of this enzyme can be safely and effectively leveraged as a therapeutic strategy.

## Data availability

All data presented are contained within the manuscript.

## Conflict of interest

The authors declare that they have no conflicts of interest with the contents of this article.
